# Comprehensive analysis of genetic risk loci uncovers novel candidate genes and pathways in the comorbidity between depression and Alzheimer’s disease

**DOI:** 10.1038/s41398-024-02968-y

**Published:** 2024-06-11

**Authors:** Bente M. Hofstra, Martien J. H. Kas, Dineke S. Verbeek

**Affiliations:** 1 Department of Genetics, University Medical Center Groningen, University of Groningen, Groningen, the Netherlands; 2 Groningen Institute for Evolutionary Life Sciences, University of Groningen, Groningen, the Netherlands

**Keywords:** Neuroscience, Genomics

## Abstract

There is growing evidence of a shared pathogenesis between Alzheimer’s disease and depression. Therefore, we aimed to further investigate their shared disease mechanisms. We made use of publicly available brain-specific eQTL data and gene co-expression networks of previously reported genetic loci associated with these highly comorbid disorders. No direct genetic overlap was observed between Alzheimer’s disease and depression in our dataset, but we did detect six shared brain-specific eQTL genes: *SRA1*, *MICA*, *PCDHA7, PCDHA8, PCDHA10* and *PCDHA13*. Several pathways were identified as shared between Alzheimer’s disease and depression by conducting clustering pathway analysis on hippocampal co-expressed genes; synaptic signaling and organization, myelination, development, and the immune system. This study highlights trans-synaptic signaling and synaptoimmunology in the hippocampus as main shared pathomechanisms of Alzheimer’s disease and depression.

## Introduction

Depression is a psychiatric disorder that affects ~10% of the general population [1]. The disease has a major impact on quality of life and often leads to occupational dysfunction as well as social isolation [2]. Patients suffer from loss of energy, impaired cognitive function, and anhedonia. These symptoms are also observed in a wide variety of neurological diseases, including Alzheimer’s disease (AD). AD affects over 35 million people worldwide [3], this number is thought to increase to 87 million by 2050 [4]. Depressive symptoms are reported to have the biggest impact on the quality of life of AD patients [5].

Both diseases are shown to be highly co-morbid [6] and are multifactorial in its origin where environmental as well as genetic factors play a role. Early life stress (ELS) due to childhood abuse or neglect seems to make the brain susceptible to depressive symptoms in humans, and ELS also causes depressive-like symptoms in rodents [7, 8]. Other depression risk factors include stressful events later in life, such as divorce or the death of a loved one. Both early or later life stress and depression are also major risk factors for AD [9, 10]. Notably, APP/PS transgenic mice recapitulating AD in humans showed depressive-like behavior, even before the onset of AD-pathology and symptoms [11]. Taken together, this points to a shared pathology, rather than depression being a mere consequence of AD.

A decrease in hippocampal volume is observed in both depressive and AD cases [12, 13]. In depression, alterations in the width of the synaptic cleft and number of hippocampal post-synaptic terminals are reported [14, 15]. This likely translates into changes in hippocampal plasticity. Plasticity in the hippocampus plays an important role in regulating reward systems [16]. Dysregulation of these reward systems can cause anhedonia seen in depressive and in AD patients [16, 17]. In AD, hippocampal involvement is widely accepted because of its volumetric changes as well as its crucial role in memory formation [18]. The loss of synaptic terminals in strongly correlated with cognitive decline in AD [19]. However, the specific subtypes of affected spines and synapses in the hippocampus seem to differ from case to case [20]. Overall, these studies further drive the hypothesis of a shared pathology in the hippocampus.

Extensive genetic research, including GWAS and their meta-analyses have identified many genetic risk factors for both diseases. These studies pointed to associated loci/genes involved in immunity, lipid metabolism, tau binding proteins, and amyloid precursor protein (APP) metabolism in AD [21], whereas loci/genes involved in calcium signaling, dopaminergic neurotransmission, glutamate neurotransmission, and presynaptic vesicle trafficking were reported to be involved in depression [22]. Notably, a shared genetic etiology underling AD and major depressive disorder was recently described [23], and Gaussian mixed modeling revealed genetic overlap between depression and AD [24]. The corresponding genes pointed to a shared role for immune response and regulation of endocytosis in AD and depression. Although these studies were informative, no distal relationships between GWAS SNPs and genes such as eQTL analysis and pathway enrichment in a relevant tissue such as brain was performed.

Therefore, in this study, we continued to explore the shared genetic background of depression and AD. We used available GWAS SNPs of depression and AD from the GWAS catalog, recently publicly available brain-specific eQTL data, and a hippocampal gene co-expression network [25]. Synaptoimmunology and trans-synaptic signaling were identified as converging biological pathways between depression and AD.

## Methods

### Collection of publicly available genetic data

Genetic loci associated with AD and depression were collected from the GWAS catalog [26] in November 2019. SNPs were selected when annotated for the traits ‘Alzheimer’s disease’ (EFO_0000249) and ‘Unipolar depression’ (EFO_0003761), respectively. We selected SNPs with *p* < 5 × 10^−5^. Studies were then filtered to maintain those SNPs annotated with ‘Alzheimer’s disease’ for AD. For depression we maintained the traits ‘Depression’, ‘Major Depressive disorder’, ‘Major Depressive disorder (broad)’ and ‘Major Depressive disorder (unexposed to adversity)’. Data exploration and workflow diagram creation were done in R studio (4.0.2).

### Collection of publicly available brain eQTL data

We extracted publicy available eQTL data (July 2020) for the 112 SNPs selected for AD and the 592 SNPs selected for depression from the recently published MetaBrain study [25]. At that time, MetaBrain comprised 2,970 brain samples from individuals of European descent and included datasets from the AMP-AD consortium [27] (AMP-AD MAYO, ROSMAP and MSBB), Braineac [28], the PsychENCODE consortium [29] (Bipseq, BrainGVEX, CMC, GVEX, LIBD, and UCLA_ASD), NABEC [30], TargetALS [31], the GTEx database [32], and the European Nucleotide Archive.

### Data simulation

Data was simulated in R studio (4.0.2) in order to find the relevance of the overlap between the AD and depression eQTL genes. All genes that exhibit eQTL effects were downloaded from the 2020-05-26 release of Metabrain [25]. 101 and 487 random genes were taken from this dataset using the replicate function to represent the AD and depression list respectively. This was repeated 1000 times for each set. Jaccard index was calculated using the following formula: ${Jaccard\; index}=\,\frac{{overlap\; set\; A\; and\; B}}{{length\; setA}\,+\,{lengh\; set\; B}}$. This was repeated for all possible combinations, totaling 1,000,000 comparisons.

### Collection of hippocampal co-expressed genes

We obtained networks of genes co-expressed with AD and depression eQTL genes in hippocampus specifically (kindly provided by HJ Westra and L Franke) [25]. This data, totaling 192 samples, was derived from FastQ files of hippocampus samples of Braineac [28], the PsychENCODE consortium [29] (Bipseq, BrainGVEX, CMC, GVEX, LIBD, and UCLA_ASD) and GTEx [32]. Genes were considered co-expressed with a Bonferroni-corrected *p* value < 0.05.

### Pathway analysis

We conducted pathway analysis in Metascape and the analysis parameters were set to the default parameters of the express analysis [33]. Annotations were extracted from the KEGG [34], GO [35], and Reactome [36] databases. Metascape grouped all pathways with a similarity of more than 30% into one cluster and adopted the name of the most significant term (i.e. biological pathway) [33]. This clustered data was exported to Cytoscape (V 3.8.0), and the figure was finalized in Adobe Illustrator (V 24.2.3).

## Results

### Compilation of lists of GWAS SNPs for depression and AD

**Fig. 1:**
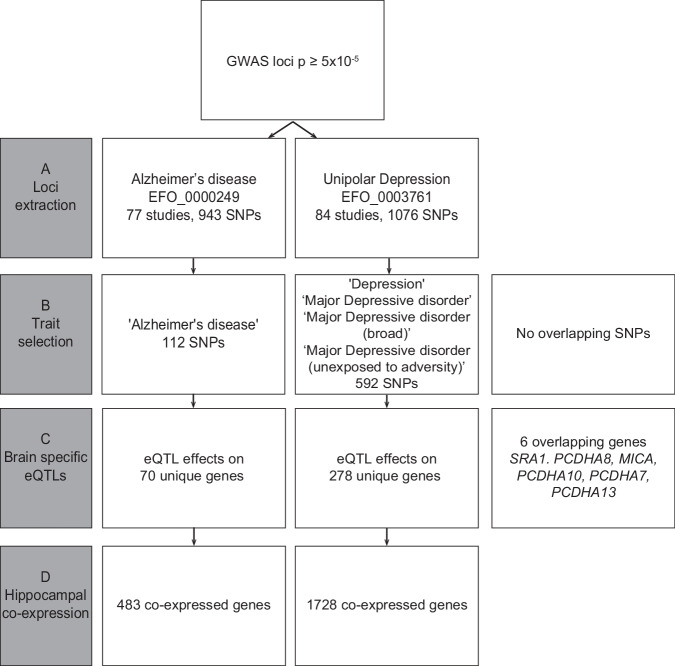
Schematic representation of the collection of the gene sets. The gene set collection consisted of four stages: **A** genetic loci were extracted from the GWAS catalog [30], **B** traits were selected to maintain only relevant studies based on criteria mentioned in the Materials and Methods, **C** brain-specific eQTL data for selected GWAS SNPs were extracted from the MetaBrain study [32], and **D** genes co-expressed with the eQTL genes in hippocampus were obtained.

To further uncover the shared genetic background between depression and AD, we first generated lists of genetic risk loci for both disorders using publicly available data from the GWAS catalog (accessed on February 7, 2019) [26]. For Alzheimer’s disease (EFO_0000249), 77 studies that reported 943 SNPs were extracted (Fig. 1A), and we selected SNPs suggestively associated with the trait Alzheimer’s disease, leading to 112 unique SNPs with a reported *p* value < 10^−5^ (Fig. 1B). The search term Uni polar depression (EFO_0003761) yielded 84 studies that reported 1076 associated SNPs. Upon selection for the traits Depression, Major depressive disorder, Major depressive disorder (broad), and Major depressive disorder (unexposed to adversity), 592 unique SNPs with a reported *p* value < 10^−5^ remained (Fig. 1B). No identical SNPs were found in the lists of GWAS risk loci for the two disorders.

### Brain-specific eQTL data reveal putative novel AD and depression risk genes

Secondly, we studied available brain-specific eQTL data for the GWAS SNPs selected for AD and depression [25]. GWAS-associated SNPs/risk loci are often noncoding and exert transcriptional effects on one or more genes, which do not have to be the nearest genes [37]. We thus collected both *cis*- and *trans*-eQTL data, as regulatory elements can be in relative proximity (<1 megabases (MB)) of their target gene (*cis*) or relatively distant (>5MB) (*trans*). For the 112 risk loci linked to AD, we collected 101 SNP–gene pairs (100 *cis*- and 1 *trans*-eQTL) corresponding to 70 unique genes (false discovery rate (FDR) threshold < 0.05) (Fig. 1C and Supplementary table S1). For the 592 risk loci associated with depression, we collected 487 SNP–gene pairs (428 *cis* and 59 *trans*-eQTLs) corresponding to 278 unique genes (FDR threshold < 0.05) (Fig. 1C and Supplementary tables S2 and S3). When we compared the genes unique for AD and depression, we identified six genes shared between both disorders: *SRA1, MICA, PCDHA8, PCDHA10, PCDHA7*, and *PCDHA13* (Supplementary fig. S1). The Jaccard index of these data was 0.01020. 1000 data sets were simulated for AD and compared to 1000 simulated datasets for depression, totaling 1,000,000 comparisons. This simulated date averaged a Jaccard index of 0.0049. Only 2.6% of the 1,000,000 simulated comparisons had greater overlap and as such the overlap in the AD and depression dataset is unlikely to be found by chance.

Despite that the biological relevance of the six shared genes needs to be interpreted carefully, the strongest significant cis-eQTL effects in the AD dataset were on *CR1* (rs3818361, *p* = 6.14 × 10^−139^; rs6656401, *p* = 2.35 × 10^−137^), ZNF284 (rs2061333, *p* = 2.79 × 10^−25^), *CR2* (rs3818361, *p* = 8.13 × 10^−23^), *IL17RC* (rs1552244, *p* = 1.03 × 10^−21^), and *MRPL39* (rs2298369, *p* = 6.39 × 10^−21^) (Table 1 and Supplementary table S1). Only one significant AD trans-eQTL was detected for *AFF3* (rs6857, *p* = 3.25 × 10^−07^). The eQTL analysis data revealed 48 genes regulated by AD SNPs that were not yet in the GWAS catalog that mainly reports genes by proximity analysis (Table 1 and Supplementary table S1).

**Table 1:** Top *cis*- and *trans*-eQTL effects of depression- and Alzheimer’s-associated SNPs.

HGNC gene name	*P* value	SNP name	Probe name	SNP type	Allele assessed	Overall Z score	Chr.	Base position	FDR	Found InProx
*AD cis-eQTL*										
*CR1*	6.14 × 10^−139^	rs3818361	ENSG00000203710.11	G/A	A	25.0917515	1	207,611,623	0	Y
*CR1*	2.35 × 10^−137^	rs6656401	ENSG00000203710.11	G/A	A	24.9462086	1	207,518,704	0	Y
*ZNF284*	2.79 × 10^−25^	rs2061333	ENSG00000186026.7	T/C	C	−10.3884106	19	44,110,055	0	N
*CR2*	8.13 × 10^−23^	rs3818361	ENSG00000117322.17	G/A	A	9.8329847	1	207,611,623	0	Y
*IL17RC*	1.03 × 10^−21^	rs1552244	ENSG00000163702.20	A/G	G	−9.5738314	3	10,093,893	0	N
*MRPL39*	6.39 × 10^−21^	rs2298369	ENSG00000154719.13	C/G	C	−9.383293	21	25,583,969	0	Y
*CR2*	1.06 × 10^−19^	rs6656401	ENSG00000117322.17	G/A	A	9.082746	1	207,518,704	0	Y
*MUC6*	4.68 × 10^−16^	rs10794342	ENSG00000184956.16	C/T	C	8.1197985	11	924,904	0	N
*CEACAM19*	2.52 × 10^−15^	rs8103315	ENSG00000186567.13	C/A	A	7.912609	19	44,750,911	0	Y
*SRA1*	6.67 × 10^−15^	rs11168036	ENSG00000213523.10	T/G	T	−7.7904846	5	140,327,854	0	N
*Dep cis-eQTL*										
*GSDME*	6.07 × 10^−123^	rs2721811	ENSG00000105928.16	A/G	G	23.5809379	7	24,709,810	0	Y
*PCDHA8*	7.23 × 10^−88^	rs3806843	ENSG00000204962.6	T/C	T	19.8713492	5	140,832,953	0	N
*PCDHA13*	1.45 × 10^−71^	rs3806843	ENSG00000239389.8	T/C	T	−17.888582	5	140,832,953	0	N
*PCDHA10*	8.73 × 10^−61^	rs3806843	ENSG00000250120.8	T/C	T	16.4477652	5	140,832,953	0	N
*ZSCAN31*	4.47 × 10^−58^	rs853679	ENSG00000235109.7	C/A	A	16.0652087	6	28,329,086	0	Y
*GMPPB*	4.76 × 10^−54^	rs13084037	ENSG00000173540.12	A/G	G	15.4796759	3	49,176,633	0	N
*AMT*	1.48 × 10^−52^	rs13084037	ENSG00000145020.15	A/G	G	−15.2568199	3	49,176,633	0	Y
*PCDHA7*	2.18 × 10^−46^	rs3806843	ENSG00000204963.6	T/C	T	14.3005182	5	140,832,953	0	N
*AREL1*	5.42 × 10^−46^	rs1045430	ENSG00000119682.17	T/G	T	14.2368404	14	74,663,532	0	Y
*SPPL3*	4.62 × 10^−43^	rs3213572	ENSG00000157837.16	G/A	A	−13.7568749	12	120,767,275	0	Y
*AD trans-eQTL*										
*AFF3*	3.25 × 10^−07^	rs6857	ENSG00000144218.19	C/T	T	−5.108585	19	44,888,997	0	N
*Dep trans-eQTL*										
*NPR3*	6.24 × 10^−15^	rs3823612	ENSG00000113389.16	G/C	C	7.7991773	7	12,219,129	0	N
*NPR3*	1.26 × 10^−14^	rs2043539	ENSG00000113389.16	G/A	A	7.7098298	7	12,214,254	0	N
*NPR3*	5.05 × 10^−12^	rs10950398	ENSG00000113389.16	G/A	A	6.9043607	7	12,225,245	0	N
*SMAD4*	5.73 × 10^−11^	rs10950398	ENSG00000141646.13	G/A	A	6.5507623	7	12,225,245	0	N
*DSTYK*	1.27 × 10^−10^	rs10950398	ENSG00000133059.17	G/A	A	6.4302364	7	12,225,245	0	N
*NR4A2*	1.69 × 10^−10^	rs10950398	ENSG00000153234.14	G/A	A	-6.3868337	7	12,225,245	0	Y
*KCND3*	3.56 × 10^−10^	rs2043539	ENSG00000171385.9	G/A	A	6.2725143	7	12,214,254	0	N
*KCND3*	5.65 × 10^−10^	rs3823612	ENSG00000171385.9	G/C	C	6.1999724	7	12,219,129	0	N
*CHRNA2*	9.22 × 10^−10^	rs10950398	ENSG00000120903.13	G/A	A	6.1224055	7	12,225,245	0	N
KCND3	3.33 × 10^−09^	rs10950398	ENSG00000171385.9	G/A	A	5.9146398	7	12,225,245	0	N

The 10 most significant SNP–gene interactions extracted from MetaBrain [32] are shown. FoundInProx refers to the identification of the gene through classical GWAS and proximity analysis. Note that genes annotated No (N) in this column might have previously been implicated in either disease but through other means. *Dep* Depression.

The *cis*-eQTL data for depression GWAS SNPs contained 428 significant SNP–gene interactions, with the top effects on *GSDME* (rs2721811, *p* = 6.07 × 10^−123^), *PCDHA8* (rs3806843, *p* = 7.23 × 10^−88^), *PCDHA13* (rs3806843, *p* = 1.45 × 10^−71^)*, PCDHA10* (rs3806843, *p* = 8.73 × 10^−61^) and *ZSCAN32* (rs853679, *p* = 4.47 × 10^−58^) (Table 1). Of these *cis*-eQTLs, 180 genes were not reported by proximity analysis (Supplementary table S2). In the *trans*-eQTL data, 59 unique significant SNP–gene interactions were present, with top effects on *NPR3* (rs3823612, *p* = 6.24 × 10^−15^; rs2043539, *p* = 1.26 × 10^−14^; rs10950398, *p* = 5.05 × 10^−12^), *SMAD4* (rs10950398, *p* = 5.73 × 10^−11^), and *DSTYKI* (rs10950398, *p* = 1.27 × 10^−10^). Of these 59 *trans*-eQTL effects, 31 were not reported using proximity analysis (Table 1 and Supplementary table S3).

### Hippocampal gene co-expression networks expose biological pathways for AD and depression

Thirdly, we obtained networks of genes co-expressed with the eQTL genes for both disorders in hippocampus specifically (Fig. 1D). The hippocampal co-expression networks were kindly provided by the MetaBrain study [25]. Following the *guilt-by-association* principle 483 genes for AD and 1728 genes for depression were present in the disease-specific hippocampal networks. The network for AD contained three gene modules and for depression contained eight gene modules (Supplementary figs. S2 and S3, Supplementary table S4).

To gain more insight into the biological functions of these gene modules, enrichment analysis on these gene modules was performed. For AD, module 1 (120 genes) was most significantly enriched for myelination (GO:0042552, *p* = 8.13 × 10^−09^) and module 2 (77 genes) showed enrichment for signaling by receptor tyrosine kinases (R-HSA-9006934, *p* = 1.26 × 10^−06^) (Supplementary figure S2, table S4). Module 3 (75 genes) also showed enrichment for myelination (GO:0042552, *p* = 8.71 × 10^−08^). For depression, module 1 (417 genes) was most significantly enriched for genes involved in trans-synaptic signaling (GO:0099537, *p* = 2.40 × 10^−70^), whereas module 2 (306 genes) was enriched for ensheathment of neurons (GO:0007272, *p* = 9.55 × 10^−25^) (Supplementary figure S3, table S4). Additionally, module 3 (174 genes) was enriched for immune-related processes, most significantly for myeloid leukocyte activation (GO:0002274, *p* = 7.08 × 10^−48^). The most significantly enriched clusters for the remainder of the modules for depression were module 4 (172 genes) oxidative phosphorylation (KO:ko00190, *p* = 8.71 × 10^−78^), module 5 (148 genes) cilium movement (GO:0003341, *p* = 3.89 × 10^−54^), module 6 (111 genes) behavior (GO:0007610, *p* = 2.63 × 10^−10^), module 7 (18 genes) NGF-stimulated transcription (R-HSA-9031628, *p* = 3.39 × 10^−11^), and module 8 (15 genes) chromatin remodeling (GO:0006338, *p* = 1.29 × 10^−4^).

### Pathway clustering analysis reveals trans-synaptic signaling, cell-part morphogenesis, and brain development as overlapping pathways in depression and AD

**Fig. 2:**
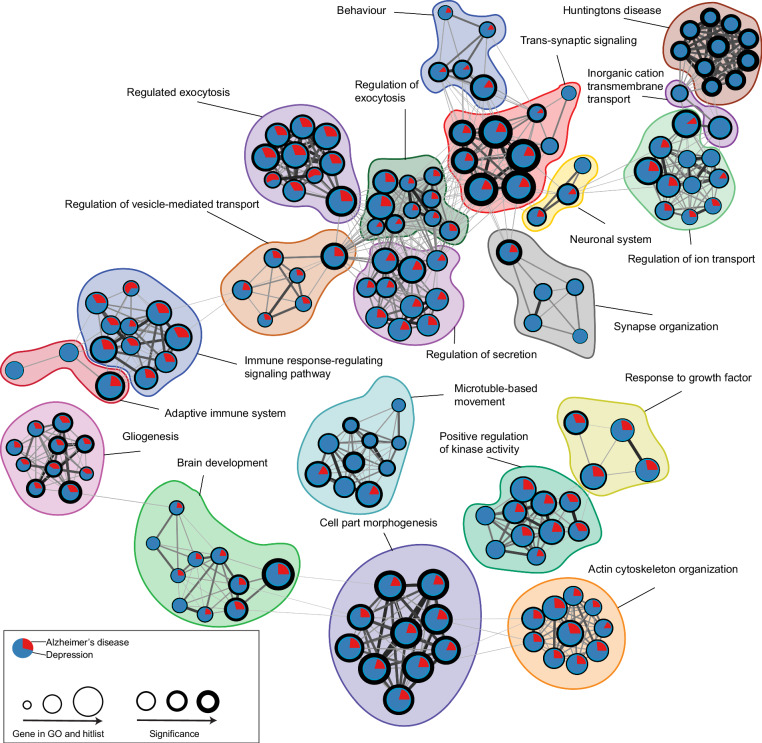
Pathway clustering analysis of depression and Alzheimer’s gene sets. Significantly enriched pathways were clustered when the similarity index >0.3, and the cluster was named by the most significantly enriched pathway. Individual pathways are represented by nodes. Pie-charts show count data for genes per gene set in that pathway. Significance is represented by the border thickness of nodes. Edges represent common genes between nodes.

Fourthly, pathway clustering was performed on all AD and depression eQTL genes, including the hippocampal co-expressed genes (483 and 1728 genes for AD and depression, respectively), of which 266 genes were shared (Supplementary table S5). We identified 20 clusters that were significantly enriched in both AD and depression datasets (Fig. 2, Supplementary table S6). These 20 clusters were manually grouped into four major categories: development (clusters brain development and cell-part morphogenesis), macrostructure (clusters synapse organization and gliogenesis), microstructure (clusters trans-synaptic signaling and inorganic cation transmembrane transport) and immune system (clusters *adaptive immune system* and immune response-regulating signaling pathway).

The cluster trans-synaptic signaling (GO:0099537) was the most significantly overrepresented in both AD and depression datasets (*p*_adj_ = 2.95 × 10^−38^). The cluster comprised 153 depression and 35 AD genes, of which 24 genes were overlapping. The next four significant clusters were *Huntington’s disease* (KEGG:ko05016, *p*_adj_ = 5.67 × 10^−35^) with 227 depression and 38 AD genes (26 overlapping), *cell-part morphogenesis* (GO:0032990, *p*_adj_ = 4.47 × 10^−32^) with 291 depression and 108 AD genes (73 overlapping), *brain development* (GO:0007420, *p*_adj_ = 4.79 × 10^−30^) with 138 depression and 48 AD genes (27 overlapping) and synapse organization (GO:0050808, *p*_adj_ = 1.21 × 10^−25^) with 98 depression and 24 AD genes (17 overlapping) (Supplementary table S6). Other significantly enriched clusters can be found in Supplementary table S6.

## Discussion

In this study we further investigated the shared pathogenetics between depression and AD by investigating available brain-specific *cis*- and *trans*-eQTLs and hippocampus-specific gene co-expression data. Our work validated previous work and showed that pathways involved in development, synaptic communication and immune regulation are common in both diseases and likely points to an important role of hippocampal plasticity in their co-morbidity. Additionally, the brain-specific eQTL data exposed candidate risk genes that were not yet reported through proximity analysis in the GWAS catalog for both AD and depression at the time of extraction.

Despite two previous studies already showed direct genetic overlap between AD and depression [23, 24], we did not find shared GWAS SNPs for the two disorders. This could be due to several reasons, for example (1) at the time of data extraction some studies such as the work by Kunkle et al. [21] was not yet present in the GWAS catalog and (2) our trait selection (Fig. 1B) may have resulted in the loss of certain SNPs that were present in the Monereo-Sáncez and/or Lutz studies [23, 24].

Typically a threshold of *p* < 5 × 10^−8^ is used to identify genome wide significant associated loci. However, by decreasing the specificity of SNP detection from *p* < 5 × 10^−8^ to *p* < 5 × 10^−5^, we aimed additionally to also identify suggestively associated loci by increasing the sensitivity of the SNP detection. Increasing sensitivity during exploratory research is especially important when researching diseases for which the standard model of high specificity has not been proven fruitful, which is the case for both depression and AD [38]. In contrast, the tissue-specificity and size of the eQTL dataset derived from MetaBrain [25] likely increased the accuracy of the SNP–gene correlations. Combined, this likely will lead to a low risk of leaving relevant SNPs out, and a high accuracy of SNP-gene correlations.

The overlapping eQTL genes between AD and depression (*SRA1, MICA, PCDHA8, PCDHA10, PCDHA7*, and *PCDHA13*), were not yet associated with these disorders through proximity analysis nor have an established role in these diseases as of yet.

*SRA1* encoding the Steroid Receptor RNA Activator 1 works as a scaffold for proteins to repress transcription of specific genes [39]. Upon binding of activated AF1-steroid receptors (such as estrogen receptor alpha (ERα)), SRA1 and its complex dissociate from the DNA, enabling transcription [40]. Therefore, changes in *SRA1* expression may contribute to sex-dependent vulnerability to developing these diseases as both depression and AD are approximately twice as prevalent in women (reviewed in [41, 42]). Besides, men with high levels of estrogen have a higher risk for depression [43]. The role of estrogen in the hippocampus seems to be in modifying synaptic plasticity, and thus dysregulation of its downstream effects through SRA1 might lead to dysregulated synaptic plasticity [44–48].

*MICA* encodes a non-classical MHC class I molecule that, in contrast to the classical MHC molecules, is not involved in antigen presentation [49]. Upon stress, MICA is expressed on the cell surface, inducing cell death [50, 51], and was detectable in post-mortem hippocampal tissue of AD as well as non-AD samples [52]. Besides, MICA seems to play a role in myelination [53] and white-matter involvement is well documented for both depression and AD [54–56]. Concordant, myelination was also present in our pathway enrichment analysis of the hippocampal gene co-expression networks (Supplementary table S4) and may further point to myelination involvement in the shared pathogenesis of AD and depression. MICA might also be involved in synaptic plasticity as classical MHC class I molecules, which have structural similarities to MICA, have also been reported to regulate brain development and synaptic plasticity (reviewed in [57]). Overall, MICA could be an interesting new shared target for future research on the shared pathogenesis, e.g. in the synaptic plasticity in hippocampus, in depression and AD.

The eQTL data for *PCDHA7, PCDHA8, PCDHA10* and *PCDHA13* need to be interpreted with caution as the *PCDHA* gene family is organized in a so-called gene cluster. This may have caused difficulties for alignment of the respective RNAseq reads to the correct gene in this cluster. Unfortunately, we did not do the analysis ourselves and could not check if this was the case. Nevertheless, the whole gene cluster is implicated in the distribution and axonal outgrowth of serotonergic neurons, which is imperative for proper serotonergic innervation [58]. Lower levels of serotonin, as well as a decreased number of serotonergic neurons, were observed in post-mortem brains of AD cases, and the serotonin levels correlated with cognitive ability [59, 60]. Knock-out of specific *Pcdha* isoforms lowered serotonin levels in the hippocampi of mice and increased depression-associated behaviors [61]. Selective serotonin reuptake inhibitors (SSRIs) are the most prescribed class of anti-depressants and, although effective in some, do not work for all patients. Interestingly, SSRI-nonresponsive patients showed decreased expression of *PCDHA6* and *PCDHA8* [62]. Combining our data with these studies, we hypothesize that these patients do not benefit from decreased serotonin reuptake since the innervating serotonergic synapses are lacking and/or not located correctly. Additionally, *PCDHA* methylation was shown to be vulnerable to environmental factors such as early life stress, a known risk-factor for both depression and AD [8, 9, 63]. Given its role in several overlapping pathways, its aberrant expression in both AD and depressive patients and its epigenetic regulation upon environmental activation, the *PCDHA* gene cluster seems a promising candidate locus in the comorbidity between the two diseases and warrants further investigation. Although only 2.6% of the simulated data showed higher overlap indexes, the shared eQTL genes should be further functionally investigated in follow-up studies to conform their role in the shared pathogenesis of AD and depression.

The most significant enriched shared pathway in our study, synaptic communication, has been shown to strengthen or weaken neuronal networks [64]. In the hippocampus, this process is imperative for new memory formation, which is well known to be impaired in AD and was more recently connected to depression [14, 65, 66]. Loss of synaptic activity and of synapses was shown to correlate with cognitive impairment to a higher degree than Aβ plaques or tau pathology [67]. Even though these are classical hallmarks of AD [13] and are often used to score disease severity. This may indicate that synaptic activity is a major player in the disease pathogenesis of AD. Besides, aberrant communication within the hippocampus is associated with decreased memory formation in depressive patients and with anhedonia in AD patients [16, 68, 69].

The shared immune system component together with the overlapping eQTL effect on *MICA*, may suggest a role for synaptoimmunology in the shared pathogenesis. This process tightly regulates synaptic signaling under control of components of the immune system [70]. For example, cytokines produced by immune cells affect the potentiation of neurons and neuronal activity in the hippocampus [71, 72] and synaptic MHC class I proteins can influence neuronal potentiation and synaptic density [57, 73, 74]. Although a link for the immune system in the etiologies of depression and AD is known (reviewed in [75]), this is not yet the case for synaptoimmunology. However, we cannot rule out that the immune system might work in parallel to disrupted hippocampal plasticity in the shared pathogenesis between AD and depression.

Interestingly, the hippocampus is one of only three areas where adult development in the form of neurogenesis is described in the human brain [76], which is seemingly consistent with our observation for a role for cell-part morphogenesis including neurogenesis and brain development in our clustering analysis. Hippocampal neurogenesis is a mechanism to cope with stress [77], and early life stress events decrease neurogenesis immediately thereafter, but also impair the neurogenic response to stress later in life [78, 79]. Changes in neurogenesis that may be the consequence of an early life stress event have been reported in the brains of AD patients, even in the early stages of the disease [80]. Taken together, increasing neurogenesis specifically in the hippocampus may open a new avenue for therapeutic development for both depression as well as AD [81, 82]. Indeed, pro-neurogenic therapies have recently been suggested for AD (reviewed in [83]), and clinical trials assessing the effectiveness of pro-neurogenic agents in depressive patients have shown positive results [84].

In conclusion, our study further points to a shared pathogenesis between AD and depression in the hippocampus and showed that the overlap in pathomechanisms between depression and AD is multi-faceted. Although our work did not rule out the contribution of other brain regions in this shared pathogenesis, the shared pathogenesis between AD and depression is seemingly due to changes in synaptic signaling in the hippocampus and/or aberrant synaptoimmunology and neurogenesis.

Supplementary information is available at TP’s website

### Supplementary information

Supplementary material overview

Supplementary figure 1

Supplementary figure 2

Supplementary figure 3

Supplementary table 1

Supplementary table 2

Supplementary table 3

Supplementary table 4

Supplementary table 5

Supplementary table 6

## Data Availability

GWAS SNP data is publicly available through the GWAS catalog [26] (https://www.ebi.ac.uk/gwas/). eQTL data is publicly available through MetaBrain [25] (https://www.metabrain.nl/).
