# Supplementary material for: Comprehensive analysis of genetic risk loci uncovers novel candidate genes and pathways in the comorbidity between depression and Alzheimer’s disease
Source: Transl Psychiatry. 2024 Jun 11;14:253. doi: 10.1038/s41398-024-02968-y (PMC11166962; doi:10.1038/s41398-024-02968-y)
Supplement: Supplementary file 1 — Supplementary material overview [file 41398_2024_2968_MOESM1_ESM.docx]

**Supplementary materials:**

Adobe Illustrator Artwork 22.0 (.ai)

Supplementary figure 1.ai

**Figure S1 Gene-gene co-expression network of Alzheimer’s eQTL genes in hippocampus**

We defined a module as containing at least 3 or more nodes defined as genes with 2 or more interactions (gene- gene co-expression effects). Such nodes are represented in yellow. Nodes with 2 or less interactions are represented in blue.

Supplementary figure 2.ai

**Figure S2 Gene-gene co-expression network of depression eQTL genes in hippocampus**

We defined a module as containing at least 3 or more nodes defined as genes with 2 or more interactions (gene- gene co-expression effects). Such nodes are represented in yellow. Nodes with 2 or less interactions are represented in blue.

Supplementary figure 3.ai

**Figure S3 Brain specific eQTL effects of overlapping genes *SRA1* and *MICA* in individual datasets extracted from Metabrain. A** eQTL effect of AD risk allele T on *SRA1* expression **B** eQTL effect of AD risk allele C on *MICA* expression **C** eQTL effect of depression risk allele T on *SRA1* expression **D** eQTL effect of depression risk allele T on *MICA* expression

Supplementary table 1.xlsx:

**Table S1 The complete list of all significant cis-eQTL SNP-gene interactions for the Alzheimer’s loci**

Supplementary table 2.xlsx:

**Table S2 The complete list of all significant cis-eQTL SNP-gene interactions for the**

**depression loci**

Supplementary table 3.xlsx:

**Table S3 The complete list of all significant trans-eQTL SNP-gene interactions for the depression loci**

Supplementary table 4.xlsx:

**Table S4 Detailed overview of the modules presented in figure S1 including gene lists and their enriched pathways**

Supplementary table 5.xlsx :

**Table S5 Gene specific pathway annotation, origin and presence in clustered pathways**

Supplementary table 6.xlsx :

**Table S6 Detailed overview of pathway clusters, their origin and the contained genes**
